# Microscopies Enabled by Photonic Metamaterials

**DOI:** 10.3390/s22031086

**Published:** 2022-01-30

**Authors:** Yanyu Xiong, Nantao Li, Congnyu Che, Weijing Wang, Priyash Barya, Weinan Liu, Leyang Liu, Xiaojing Wang, Shaoxiong Wu, Huan Hu, Brian T. Cunningham

**Affiliations:** 1 Department of Electrical and Computer Engineering, University of Illinois at Urbana-Champaign, Champaign, IL 61822, USA; yanyux2@illinois.edu (Y.X.); nantaoli@illinois.edu (N.L.); pbarya2@illinois.edu (P.B.); weinanl3@illinois.edu (W.L.); leyangl2@illinois.edu (L.L.); 2 Holonyak Micro and Nanotechnology Laboratory, Champaign, IL 61822, USA; congnyu2@illinois.edu (C.C.); weijing4@illinois.edu (W.W.); wang535@illinois.edu (X.W.); 3 Department of Bioengineering, University of Illinois at Urbana-Champaign, Champaign, IL 61822, USA; 4 Carl R. Woese Institute for Genomic Biology, Urbana, IL 61801, USA; 5 Zhejiang University-University of Illinois at Urbana-Champaign Institute, International Campus, Zhejiang University, Haining 314400, China; shaoxiongwu@intl.zju.edu.cn (S.W.); huanhu@intl.zju.edu.cn (H.H.); 6 State Key Laboratory of Fluid Power & Mechatronic Systems, Zhejiang University, Hangzhou 310027, China; 7 Cancer Center at Illinois, Urbana, IL 61801, USA

**Keywords:** photonic metamaterials, microscopy, plasmonic, photonic crystals, label-free, fluorescence, biomolecular detection, biosensor

## Abstract

In recent years, the biosensor research community has made rapid progress in the development of nanostructured materials capable of amplifying the interaction between light and biological matter. A common objective is to concentrate the electromagnetic energy associated with light into nanometer-scale volumes that, in many cases, can extend below the conventional Abbé diffraction limit. Dating back to the first application of surface plasmon resonance (SPR) for label-free detection of biomolecular interactions, resonant optical structures, including waveguides, ring resonators, and photonic crystals, have proven to be effective conduits for a wide range of optical enhancement effects that include enhanced excitation of photon emitters (such as quantum dots, organic dyes, and fluorescent proteins), enhanced extraction from photon emitters, enhanced optical absorption, and enhanced optical scattering (such as from Raman-scatterers and nanoparticles). The application of photonic metamaterials as a means for enhancing contrast in microscopy is a recent technological development. Through their ability to generate surface-localized and resonantly enhanced electromagnetic fields, photonic metamaterials are an effective surface for magnifying absorption, photon emission, and scattering associated with biological materials while an imaging system records spatial and temporal patterns. By replacing the conventional glass microscope slide with a photonic metamaterial, new forms of contrast and enhanced signal-to-noise are obtained for applications that include cancer diagnostics, infectious disease diagnostics, cell membrane imaging, biomolecular interaction analysis, and drug discovery. This paper will review the current state of the art in which photonic metamaterial surfaces are utilized in the context of microscopy.

## 1. Introduction

Label-free biosensors that utilize transducer surfaces that accumulate captured analytes and subsequently generate a change in the measured property always require a large number of molecules to be gathered before the signal is observable above background noise. Examples include surface plasmon resonant (SPR) sensors, photonic crystal (PC) biosensors, quartz crystal microbalances (QCM), whispering gallery mode (WGM) ring resonators, interferometric waveguides, and reflection interference devices. As the number of accumulated target molecules decreases, we reach a regime in which the captured molecules no longer resemble a semi-continuous thin film, but rather become a sparse population of individual molecules, separated by large distances dispersed over the transducer surface area, which can be hundreds of square micrometers.

Of all the biosensor transducers, only a handful are capable of generating images of the detected analytes and the associated ability to count them with digital precision, while fewer still are capable of doing so without chemical/enzymatic amplification. Localized SPR imaging is a representative example of label-free biosensor imaging on an array of plasmonic nanorods [1]. Interferometric scattering (IScat) is an imaging approach that has demonstrated capability for measuring biomolecular on/off rates through observation of single biomolecule binding/unbinding events allowing label-free imaging of biomolecules and nanoparticles as small as several nanometers in diameter [2]. Thin film interference scattering effects can assist contrast for detection of surface-attached viruses, metal nanoparticles, and macromolecules [3,4,5]. Fluorescence microscopies, including far-field epifluorescence, near-field optical spectroscopy, and super-resolution microscopy [6,7], can observe single fluorophores and quantum dots [8], using cooled image sensors with sufficiently low noise for observing small numbers of photons. Using a fluorescence microscope, contrast can be enhanced by nanostructures such as optical traps [9].

Photonic metamaterials are a unique category of matter defined by a periodic modulation of dielectric permittivity in at least one spatial dimension [10,11,12]. The operating principles behind photonic metamaterials remain a field of intense study due to the variety of resonant mode types [13,14,15,16,17,18], range of resonant wavelengths from ultraviolet [19] through infrared [20,21], large palette of materials (dielectric, metallic, semiconducting) [22], and interesting selection of lattice types [23,24]. Due to the rich set of physical principles that can be engineered to produce useful features, photonic metamaterials enable a broad set of commercially important applications that include optical reflectance/transmission filters [25,26,27], “slow light” materials [28,29], electromagnetic transparency [30,31,32], light extractors for LEDs/lasers [33,34,35], and meta-lenses [36]. When considered in the context of biological applications, photonic metamaterials have been utilized effectively for label-free detection of biomolecules and cells when a far-field resonant property (such as the reflection or transmission spectrum) is tuned by attachment of molecules, cells or viruses. The electromagnetic fields associated with the resonances of photonic metamaterial can be harnessed to amplify the electric field power experienced by light emitters (such as fluorophores or quantum dots) or by light scatterers, so as to generate greater photon output than the same material on an unpatterned glass microscope slide. At the same time, photons emitted on a photonic metamaterial will engage with their resonant dispersion characteristic to follow well-defined pathways of wavelength and exit angle, which can be used to design structures that enhance the efficiency of emitted photon capture into the numerical aperture of an objective. The electromagnetic absorption and emission properties of nano-objects are found to form “hybrids” with photonic metamaterial resonances, which have been demonstrated to modify their absorption cross-section, scattering cross-section, and lifetime [37,38,39]. Such hybrids can not only be used in the context of biodetection, but also to catalyze specific chemical reactions through the presence of reactive electrons [40]. By tailoring light–matter interactions, both dielectric and plasmonic periodical nanostructures can enable the control of absorption, emission rate, radiation pattern and ratio between various decay channels. Dielectric metamaterials usually have high quality factor (~10^3^) and low loss, but facing changelings in modest field enhancements, intensive nanofabrication, and narrow linewidth. Conventional plasmonic metamaterials, on the other hand, have high confinement and small mode volume, but have ohmic loss restriction with low radiative efficiency, low quality factor (~10), and request sub-10 nm fabrication.

An early limitation of photonic metamaterials was the necessity to fabricate features with nanometer-scale precision, with dimensions that are generally a fraction of an optical wavelength. While expensive and low-throughput approaches such as electron beam lithography may be used to generate initial prototypes of a new design, there are now several low cost and scalable approaches that can manufacture photonic metamaterials at sufficient volume and price for single-use disposable applications that are consistent with other labware used in biology research and diagnostics. While methods such as holographic lithography are capable of producing subwavelength-scale periodic spatial patterns, a much greater degree of flexibility and scaling has been obtained through nanoreplica molding upon glass or flexible plastic surfaces, enabling labware such as biosensor microplates [41,42,43,44,45].

Two additional key features enable adoption of photonic metamaterials into microscopy. First, it is important for the electromagnetic fields associated with optical resonances to extend into the liquid media that the device is in contact with, so that cells, biomolecules, and tags can easily enter an evanescent field volume where enhanced excitation and directional emission phenomena take place. Due to this requirement, photonic metamaterials used in the context of microscopy generally have an “open face” configuration as a two-dimensional nanostructured surface that does not require analytes to diffuse deeply into a three-dimensional volume. Second, the entire surface of the photonic metamaterial must be able to serve as the sensing transducer, so it is not necessary for detected analytes to diffuse toward and bind with “hot” regions that only comprise a small fraction of the surface area [46]. While the evanescent field magnitude associated with a photonic metamaterial resonance may not be spatially uniform, ideally an analyte landing anywhere will participate in resonant enhancement, which forms the basis for observing light–matter interactions with an imaging-based approach.

This review will summarize the current state of research at the intersection of photonic metamaterials and microscopy, focused upon applications in biodetection. We will begin by considering label-free imaging microscopies in which phase shifts, resonant wavelength shifts, enhanced absorption, and enhanced scattering are obtained using a photonic metamaterial to generate image contrast. Next, we will review microscopies in which a chemical or nanoparticle tag interacts with a photonic metamaterial to generate enhanced contrast. In this section, we will consider nano-objects, such as plasmonic metal nanoparticle tags, quantum dots, and fluorescent dyes. In the context of photon-emitting tags, metamaterials are used to manipulate spontaneous emission rate, emission direction, lifetime, photobleaching, and blinking. The prospects for super-resolution microscopies that take advantage of the structured electromagnetic field profiles of photonic metamaterials also will be discussed. Finally, we will summarize the existing nanofabrication technologies for metamaterials using laser-based lithography, electron/ion beam lithography, nanoimprinting, and self-assembly. 

To limit the scope of the review, we will not cover microscopies that utilize non-periodic nanostructures that do not meet the definition of a photonic metamaterial, and we will not cover photonic metamaterial-based biosensors that are not used as substrates for microscopy. Several excellent reviews on optical imaging systems based on metalens [47,48,49] and metamaterial-based biosensors [50,51,52] with bulk signal readout are provided for readers interested in these topics.

## 2. Metamaterial-Enhanced Label-Free Imaging

The light scattered from the sample carries an abundance of information regarding the structural details of the illuminated specimen. However, since most biological samples (such as cells and tissues) are near-transparent prior to staining, it is challenging to obtain satisfactory label-free images via the scattered light as the source of contrast. The enhanced light–matter interaction offered by optical metamaterials provides an excellent solution to such a dilemma, and several exemplary novel label-free imaging techniques based on metamaterials will be respectively examined in this section.

### 2.1. Metamaterial-Enhanced Phase Microscopy

**Figure 1:**
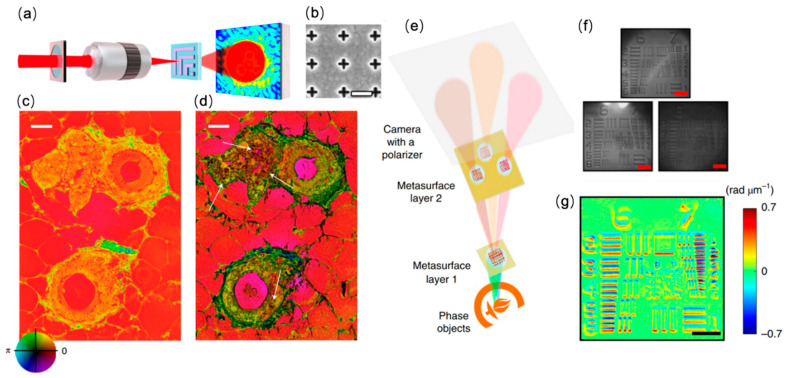
Metasurface-based phase contrast microscopy. (**a**) Schematic of the surface plasmon enhanced ptychographic phase microscope. (**b**) SEM image of the nanostructured metasurface for surface plasmon generation. Sale bar: 450 nm. (**c**) Ptychographic reconstructed phase image of an unstained tissue section. (**d**) Metasurface-enhanced ptychographic phase image of the same tissue sample shown in (**c**). Scale bar: 25 μm. Reprinted with permission from Ref. [54]. Copyright 2021 Springer Nature. (**e**) Schematic of the compact metasurface-based quantitative phase microscope. (**f**) Three differential interference contrast (DIC) images obtained by the metasurfaces. Scale bar: 50 μm. (**g**) Reconstructed phase image of the target in (**f**). Scale bar: 50 μm. Reprinted with permission from Ref. [55]. Copyright 2019 Springer Nature.

Optical phase contrast microscopy is a powerful imaging technique based on the phase difference between the reference light and the light passing through the sample of interest, and it has been widely utilized for the label-free imaging of weakly scattering samples such as cells and tissues [53]. Despite the sensitivity of conventional optical phase contrast microscopy, the image contrast for thin samples can be insufficient for visualization. By combining phase contrast microscopy with plasmonic metasurfaces, Balaur et al. developed a ptychographic phase microscopy in which silver-coated metasurfaces were utilized as the substrate for imaging (Figure 1a) [54]. By carefully designing the structure and periodicity of the unit cell (Figure 1b), the authors exploited the nonlinear optical response in both the amplitude and phase of light transmitting through the metasurfaces for the enhancement of signal contrast. Through label-free sample demonstration using both ultra-thin samples (<100 nm thick) and 4 um thick breast tissue, a substantial increase in phase contrast can be observed in comparison with conventional ptychography (Figure 1c,d).

Another application of metasurfaces in the development of phase contrast microscopy is to achieve miniaturization of the imaging system. Conventional quantitative phase imaging (QPI) techniques, despite their outstanding performance, are based on interferometry, thus requiring large and complex optical components, which can limit the applicability of QPI. By virtue of the polarization and spatial multiplexing capabilities of metasurfaces, Kwon et al. proposed a compact single-shot quantitative phase microscope in which two cascaded silicon metasurface layers were deployed to simultaneously achieve lateral shearing, phase shifting, and imaging (Figure 1e) [55]. With the cascaded metasurfaces, the fundamental limits imposed by refraction-based optics can be overcome and consequently, the overall volume of the miniaturized QPI system was reduced to 0.62 × 0.41 × 1.00 mm^3^ by integrating the two metasurfaces onto a single double-sided metamaterial. Using the metasurfaces, three differential interference contrast (DIC) images can be obtained simultaneously (Figure 1f), which can then be used to retrieve the quantitative phase information of the sample under observation (Figure 1g). The miniatured phase imaging system offers great potential for point-of-care phase imaging systems including microscopes and endoscopes.

### 2.2. Metamaterial-Enhanced Darkfield Microscopy 

**Figure 2:**
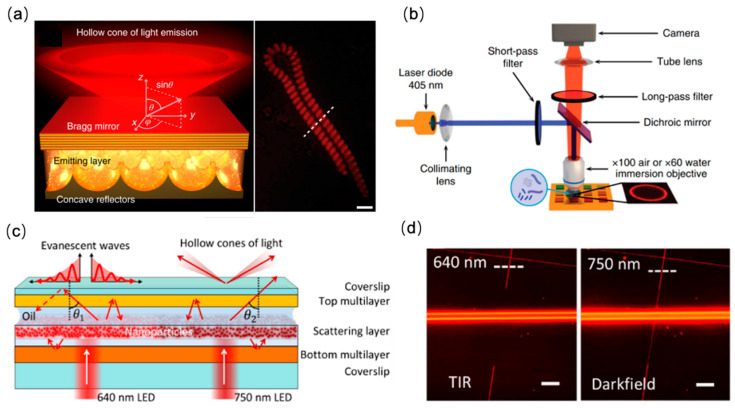
Metasurface-enabled darkfield microscopy. (**a**) Left: Schematic of the luminescent photonic substrate. Right: Darkfield image of a micro-algae on the nanophotonic substrate. Scale bar: 5 μm. (**b**) Schematic drawing of the microscope used for imaging with the luminescent surface as the imaging substrate. Reprinted with permission from Ref. [57]. Copyright 2020 Springer Nature. (**c**) Design of the planar photonic chip for darkfield illumination (at 750 nm excitation) and total internal reflection imaging (at 640 nm excitation). (**d**) Imaging demonstration of two polymer nanowires. Scar bar: 20 μm. Reprinted from Ref. [58].

Unlike phase microscopy, which has contrast that originates the sample-induced phase delay, darkfield microscopy obtains outstanding contrast by exploiting the light scattered off the specimen of interest while reducing the background noise by rejecting the incident light launched at large incident angles [56]. Conventional darkfield microscopes utilize dedicated objective lenses that have a smaller numerical aperture than the incident light cone in order to avoid collecting background light, which consequently deteriorates the spatial resolution obtainable by such darkfield microscopes. To overcome the complexity and resolution challenge imposed by conventional darkfield microscopy, recently metasurfaces were proposed as the imaging substrates that enable darkfield imaging using a simple and compact instrument configuration. As shown in Figure 2a, Chatzot et al. designed a luminescent surface by vertically integrating a Bragg mirror, light-emitting layer (consisting of CdSe/CdS quantum dots) and a micropatterned concave reflector substrate [57]. When illuminated with a laser diode at 405 nm, the quantum dots emit light with a center wavelength of 630 nm, which is confined between the concave reflector and the Bragg mirror and can only emerge at an oblique angle (as defined by the structure of the Bragg mirror), allowing for darkfield illumination. The authors demonstrated darkfield imaging of various samples using a simple bright-field optical microscope but with the luminescent metasurface as the sample substrate (Figure 2b).

Similarly, Kuai et al. designed a passive planar photonic substrate that allows for both darkfield imaging and total internal reflection (TIR) imaging at different wavelengths [58]. As shown in Figure 2c, the planar photonic chip comprises two dielectric multilayers with different photonic band structures. In place of quantum dots, the authors doped the middle layer with TiO_2_ nanoparticles to produce scattering in the propagation direction of the incident light. Confined between the two dielectric multilayers, the light will either be entirely trapped (leading to generation of an evanescent field for TIR illumination for an incident wavelength of 640 nm), or transmit at a large oblique angle that allows for darkfield illumination (for incident wavelength at 750 nm). Using a simple transmission microscope, the TIR and darkfield imaging of nanowires were demonstrated at the two wavelengths (Figure 2d). 

### 2.3. Metamaterial-Enhanced Refractometric Microscopy 

Dielectric metasurfaces based on subwavelength structures offer tunable optical resonances and strong light confinement that have been widely explored for near-field sensing and imaging [59]. Notably, high quality factor (Q-factor) metasurfaces are extremely sensitive and responsive to local refractive index (RI) changes induced by individual spatially overlapped biomolecules in surface-confined electromagnetic fields, enabling ultrasensitive label-free biosensing. Previously, a spectrometer was leveraged to monitor the resonance wavelength or the reflection intensity shift from a confined region and to sense analyte binding to the metasurfaces. More recently, the combination of high Q-factors supported by metasurfaces and imaging-based data acquisition has been studied to realize massively parallel sensing with high spatial and spectral resolution. 

**Figure 3:**
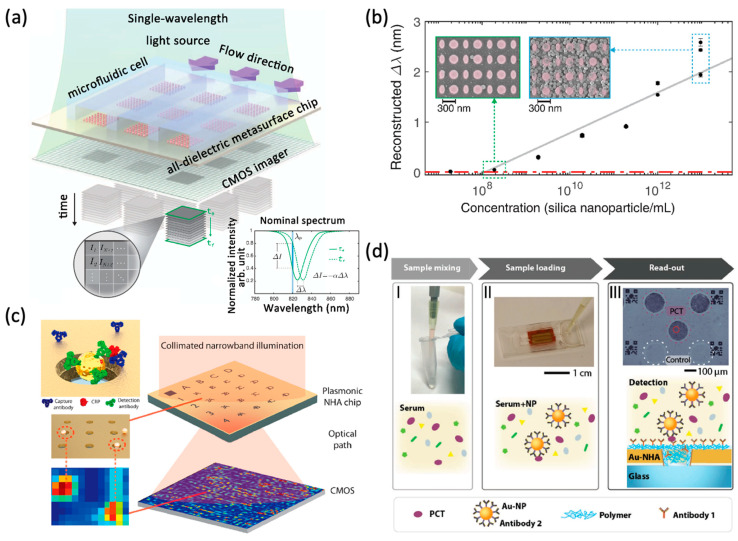
Metasurface-based refractometric microscopy for biomolecular sensing. (**a**) The single-wavelength algorithm with spectral displacement reconstruction assists the principle of imaging biosensors. A sketch of the real-time flow into the imaging platform shows a 2D microarray of all-dielectric sensors integrated with a microfluidic unit composed of three independent flow channels. Biomarker binding can be detected by tracking the resonance wavelength or by the intensity change (ΔI) at a fixed detection wavelength (*λ*p). Reprinted from Ref. [60]. (**b**) Reconstructed spectral shift (Δ*λ*) calibration curve of biotinylated silica nanoparticles. Reprinted from Ref. [60] (**c**) Nanoparticle-enhanced plasmonic imager is used to detect the antigen (red) that has been recognized by the capture antibody (blue) immobilized on the Au-NHA, and then is recognized by the detection antibody (green) attached to the Au-NPs. To achieve the purpose of detection and precise image processing, the transmission signal is displayed as an image heat map through the plasmonic NHA chip and CMOS camera, thereby realizing the digital detection of biomolecules. Reprinted from Ref. [61]. (**d**) Process of using DENIS to detect and quantify PCT and CRP, including the mixing and loading of serum samples, and the signal output through Au-NHA chip. Reprinted from Ref. [60].

Altug and colleagues have developed a spectrometer-free optofluidic biosensor based on dielectric metasurfaces utilizing single-wavelength imaging (Figure 3a). This method includes supporting a diatomic dielectric metasurface that provides a quasi-bound state in continuum (BIC) mode, allowing high-quality resonance with accessible near-fields through the destruction of in-plane symmetry. As shown in Figure 3b, the large-area metasurface chip is configured as a microarray and integrated with microfluidics on the imaging platform for real-time detection of breast cancer extracellular vesicles containing exosomes [60]. In addition, the same research group developed a variety of detection methods for related biomarkers of inflammatory diseases. They reported a bright-field imaging plasmonic biosensor that allows visualization of single subwavelength gold nanoparticles (NPs) on large-area gold nanohole arrays (Au-NHAs). The sensor generates image heatmaps that reveal the locations of single NPs as high-contrast spikes, enabling the detection of individual NP-labeled molecules (Figure 3c). They implemented the method in a sandwich immunoassay for the detection of biotinylated bovine serum albumin (bBSA) and human C-reactive protein (CRP), a clinical biomarker of acute inflammatory disease [61]. On this basis, they optimized and developed a portable biosensor based on nanoparticle-enhanced digital plasmonic imaging for rapid and sensitive detection of two sepsis-related inflammatory biomarkers from blood serum [62].

**Figure 4:**
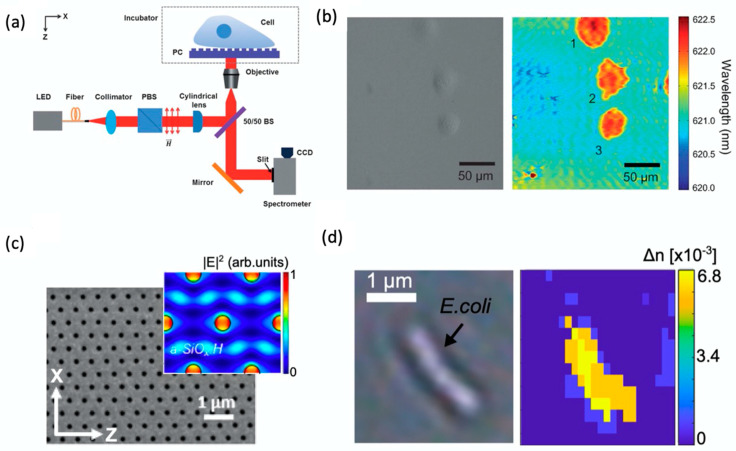
Metasurface-based refractometric label-free microscopy for cell morphology. (**a**) Schematic of the photonic-crystal-enhanced microscopy (PCEM) instrument. (**b**) Bright field (left) and PWV imaging (right) of Panc-1 cells attached to the PC surface. Reprinted with permission from Ref. [64]. Copyright 2013 The Royal Society of Chemistry. (**c**) SEM image of the dielectric nanohole array and simulated electric field at resonance in the inset. (**d**) Bright field (left) versus hyperspectral (right) imaging of an individual Escherichia coli obtained with a dielectric nanohole array. Reprinted from Ref. [68].

Apart from biomolecular detection and imaging, metasurface-based refractometric microscopy is also applied in the label-free observation of cell morphology. Photonic-crystal-enhanced microscopy (PCEM) has been demonstrated as a versatile platform for quantification and imaging of cell-surface interactions, such as stem-cell differentiation, invasion, chemotaxis, and apoptosis [63,64,65,66]. In PCEM (Figure 4a), a 1-D PC consisting of wavelength-scale gratings with high refractive index titanium dioxide (TiO_2_) or silicon nitride (Si_3_N_4_) coating is utilized as the imaging substrate, which offers subcellular spatial resolution and large field of view. When the PC is illuminated at normal incidence with a broadband light source, the resonant wavelength is reflected with nearly 100% efficiency. Cell attachment on the PC surface results in a resonant peak wavelength value (PWV) shift, which is recorded on a pixel-by-pixel basis. Chen et al. demonstrated label-free imaging of the evolution of cell attachment and morphology during chemotaxis and drug-induced apoptosis (Figure 4b) [64]. Additionally, time-lapse imaging of adhesion and migration processes of single 3T3 fibroblast cells has been demonstrated using PCEM [65]. Zhuo et al. reported the quantitative imaging of focal adhesion cluster dynamics by PCEM [66]. Furthermore, Juan-Colás and colleagues utilized PC resonant hyperspectral imaging for parallel, in vitro, real-time mapping of the secretion of a signaling protein from individual cells in a label-free manner [67].

More recently, Conteduca et al. demonstrated a dielectric nanohole array (Figure 4c) in amorphous silicon (a:Si) that supports two optical modes, both with relatively moderate Q-factors (300–400) and submicron spatial resolution, desirable for both imaging and biochemical sensing [68]. They demonstrated IgG sensing with a limit of detection (LOD) lower than 1 pg/mL using a TM mode with high Q-factor and high surface sensitivity, while the high resonance amplitude and strong confinement of the TE mode enables a spatial resolution below 1 μm that clearly resolves features of individual *E. coli* bacteria (Figure 4d). Using biochemical imaging and sensing upon a dielectric nanohole array, Conteduca et al. exploited the same principle for the detection of large extracellular vesicles (EVs), down to an LOD < 10^3^ EV mL^−1^ (<10 pM). They also utilized CD9 as a surface protein of EVs to confirm the high specificity of their approach [69]. 

### 2.4. Metamaterial-Enhanced Elastic Scattering Microscopy

**Figure 5:**
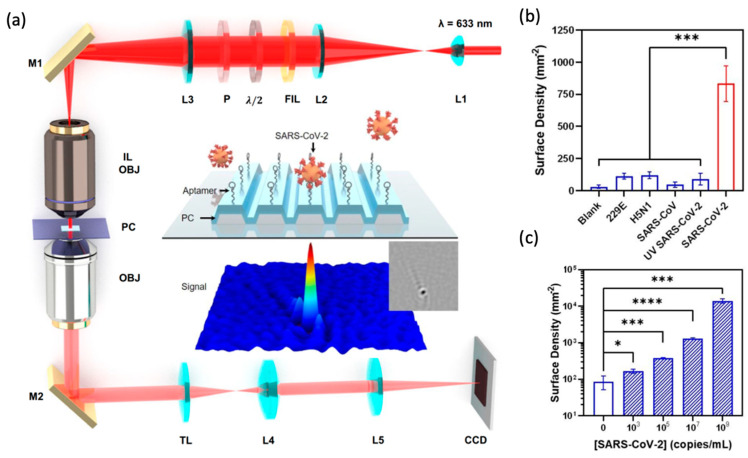
Metamaterial-Enhanced Interferometric Scattering Microscopy (**a**) Schematic of the photonic resonator interferometric scattering microscopy (PRISM) instrument. Inset: detection scheme for label-free detection of pseudotyped SARS-CoV-2 using DNA aptamers. (**b**) Selectivity and (**c**) sensitivity of the PRISM-based SARS-CoV-2 biosensor. Error bars represent the standard deviations of at least three independent measurements. (* significant; ** very significant; *** and **** extremely significant.) Reprinted from Ref. [71].

The elastically scattered photons from micro and nanoscale objects provides a universal signal for label-free microscopy. However, the scattering signal intensity decreases drastically as the size of the object shrinks, which makes observations on extremely small targets such as proteins, viruses, and nanoparticles a difficult task. The optical signals emitted from these nanoscale objects are likely to be overwhelmed by background noise, mechanical vibrations, and photon shot noise. To improve the signal-to-noise ratio, researchers have developed various methods to boost the scattering signal. One such method is surface plasmon resonance microscopy (SPRM), which uses the evanescent field on the surfaces of thin metal films to provide near-field enhancement of scattered light from nano-objects [70]. Another approach is to measure the interference signal between the scattered light from the object and the reference light from the source, known as interferometric scattering microscopy (iSCAT) [5]. In this case, one should expect a higher signal intensity compared to solely measuring the scattered light on small particles. Combining the ideas behind SPRM and iSCAT, researchers have developed photonic resonator interferometric scattering microscopy (PRISM) [71], shown schematically in Figure 5. PRISM utilizes a metasurface to enhance scattering signals while mitigating the intensity penalty of small objects by measuring the interfered light. 

Metasurfaces such as photonic crystals (PCs) enhance the scattering cross-section of micro and nano scatterers via near-field excitation. The physical picture of this interaction can be delineated by temporal coupled-mode theory (TCMT) [72,73], where the PC is treated as a resonator and is allowed to couple with the non-resonant nanoparticle (NP) antennas. The resulting scattering cross-section enhancement ratio compared to a layer of solitary gold NPs can be calculated as [73]

(1) $$\Lambda \left(\omega_{0}\right)=\frac{2\lambda_{0}\alpha }{\pi nd_{e}}\frac{\gamma_{r}}{{\left(\gamma_{r}+\gamma_{sc}\right)}^{2}}$$

where $\lambda_{0}$ is the resonant wavelength, α is the energy confinement of the PC mode in the NP layer, n is the refractive index of water and $d_{e}$ is the effective length of the evanescent field. This enhancement effect prediction exhibits good agreement with finite element method simulations [71]. Besides magnifying the scattered light, the benefits of incorporating a PC and small scatterer include improving the collection efficiency and attenuating the reference light. At resonance, the PC only allows 1% of the incident light to transmit, eliminating the need for additional measures to attenuate the reference light. Compared to the traditional iSCAT, PRISM has shown approximately 5 times greater signal contrast for detection of gold nanoparticles in the 5–30 nm diameter range [71].

Cunningham and colleagues demonstrated observation of single AuNPs, proteins [71], and virions [74] using PRISM. AuNPs with diameters down to 5 nm can be individually discerned from the background noise. Ferritin (MW = 440 kDa) and fibrinogen (MW = 340 kDa) prepared in buffer at concentrations of 100 nM can be observed with contrast ranging from −0.5% and −1. Motivated by the need for rapid, inexpensive, simple, and ultrasensitive virus detection requirements of the COVID-19 pandemic, researchers have also explored the possibility of label-free viral level determination for intact SARS-CoV-2 with PRISM through selective capture by immobilized DNA aptamers on the PC metamaterial surface (Figure 5a, inset). The targeting particles are pseudotyped SARS-CoV-2 (p-SARS-CoV-2) virions derived from lentiviral vectors with the spike proteins of SARS-CoV-2 decorated on the envelope surface. P-SARS-CoV-2 in buffer solution, with concentration as low as $1\times 10^{3}$ copies/mL, can be distinguished from the blank control. To simulate point-of-care situations, p-SARS-CoV-2 viruses are dissolved in crude human saliva, and the result presents a limit of detection of $5\times 10^{3}$ copies/mL (Figure 5b,c).

## 3. Metamaterial-Enhanced Imaging with Tags

Synthesized nanoscale materials made from metallic, magnetic, semiconducting and dielectric materials have shown broad applications in the biosensing field. Nano-particle tags, while at a similar size scale to biomolecules, can be precisely engineered to provide high surface-to-volume ratio, strong wavelength-specific interactions with electromagnetic fields, and tunable surface chemistries [75]. Exploiting the significant and versatile light-manipulation ability of metamaterials to elevate the optical processes (localized surface plasmonic resonance (LSPR), guided mode resonance (GMR), fluorescence, surface plasmon polaritons (SPP)) coupled with biological/chemical binding effects between nanoparticle tags and bio-analytes has shown promising results in providing rapid, ultrasensitive, high signal-to-noise ratio (SNR), point-of-care (PoC) and super resolution detection for biomolecules and viruses.

### 3.1. Plasmonic Nanoparticles

**Figure 6:**
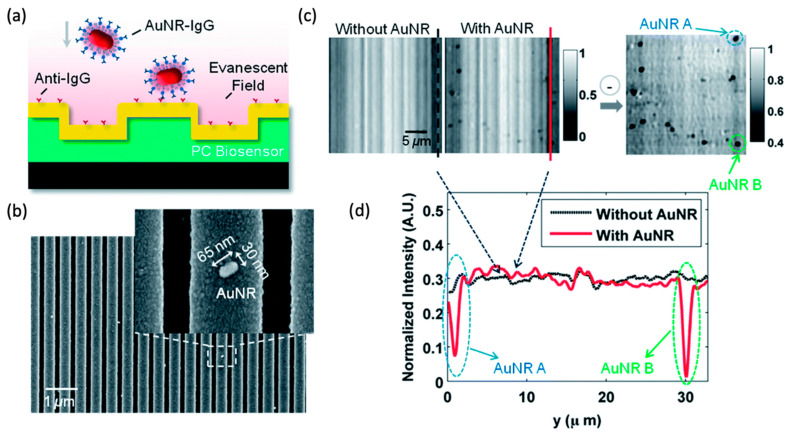
PCEM detection of protein–protein binding. (**a**) Schematic illustration of the attachment of AuNR–IgG (AuNR conjugated with SH-PEG–IgG) on a PC biosensor surface. (**b**) SEM images of AuNR–IgG attached to the PC biosensor surface. Inset: Zoomed-in image. (**c**) PCEM detected PIV images and the difference between without and with AuNR–IgG on the PC surface. (**d**) Two representative cross-section lines of the normalized intensity images with/without two AuNRs-IgG on the PC surface. Reprinted with permission from Ref. [76]. Copyright 2014 The Royal Society of Chemistry.

In comparison with dielectric nanoparticles, plasmonic nanoparticles feature much larger extinction cross-sections owing to the dissipative nature of noble metals, thus allowing for a better signal-to-noise contrast, and they are in general more favored as the tags for biosensing. Zhuo et al. first demonstrated specific detection of functionalized nanoparticle (NP) tags using photonic crystal enhanced microscopy (PCEM) [76]. The attachment of metallic on the PC is clearly reflected by the shift of reflection peak wavelength value (PWV) and peak intensity value (PIV) image, where those shifts are caused by the localized change of effective refractive index and the absorption of metal. Furthermore, this work demonstrated a sandwich-style assay by using a pre-functionalized PC surface conjugated with capture molecules. They prepared AuNP–IgG conjugates by functionalizing Au nanorods (AuNR) with SH-PEG–IgG molecules. Meanwhile, anti-IgG was immobilized onto the PC surface by exposing the PC to anti-IgG solution, as shown in Figure 6. Adding AuNR–IgG conjugates onto the functionalized PC surface resulted in specific binding to anti-IgG (SEM images shown in Figure 6b, which were detected as a PIV reduction in the AuNP area. Figure 6c shows PIV images taken before and after AuNR–IgG attachment. Two representative normalized intensity plots with/without two AuNRs-IgG on the PC surface are shown in Figure 6d. This work demonstrated that PCEM can successfully detect the intensity reduction in presence of AuNR–IgG, which laid the foundation for digital-resolution microscopy of biomolecule and virus using PC surfaces. 

**Figure 7:**
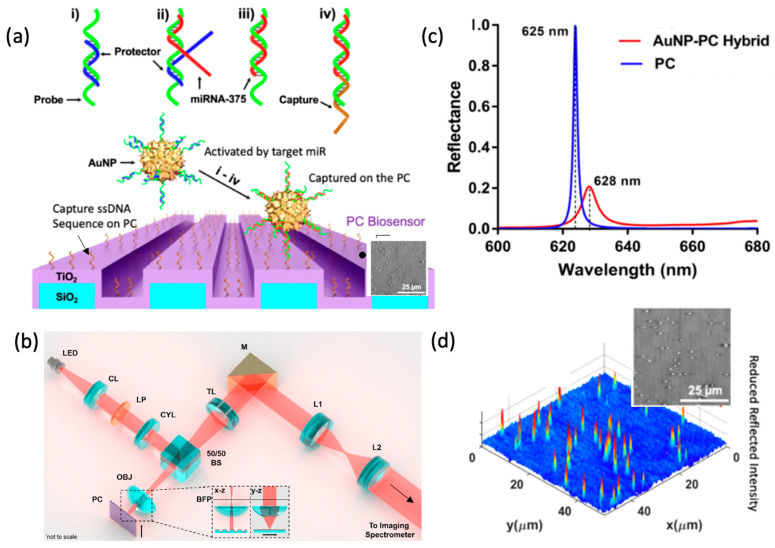
Detection of microRNA with digital-resolution and single-base selectivity by photonic resonator absorption microscopy. (**a**) Components of the toehold DNA–AuNP and miR detection by PC biosensors. (**i**) The DNA probe (green color) is bound by a protector (blue color, partially complementary) which prevents binding to the PC sensor. (**ii**–**iv**) target miR (red color) binds as toehold (**ii**), resulting in strand displacement of the protector (**iii**), which (**iv**) stabilizes probe binding to the capture DNA on the PC surface (**iv**). (**b**) PRAM instrument schematic (**c**) Simulated reflectance spectrum of the PC alone (blue) and the AuNP–PC hybrid (red). According to simulation, hybrid formation results in a reflectance peak wavelength shift (Δ*λ*) to 628 nm from 625 nm and a reflectance peak intensity drop (ΔI). (**d**) A 2D gray-scale PRAM image (Upper left) is represented in the 3D contour plot (Lower right), demonstrating the individual AuNP peak intensity shifts. Reprinted from Ref. [77].

When PCEM is used for imaging surface-attached resonantly absorbing NPs, the microscopy is called Photonic Resonator Absorption Microscopy (PRAM). Canady et al. first demonstrated PRAM by integrating AuNP tags on a PC biosensor to achieve single-step, ultrasensitive and highly selective detection of miRNA. The work used a “toehold probe” strategy, where the AuNP tags are conjugated with highly selective nucleic acid toehold probes that, upon capture of the target sequence, will result in “activation” of the AuNPs. The “activated” AuNPs will enable the remaining part of the probes to hybridize with complementary ssDNA capture molecules immobilized on the PC surface, forming a sandwich structure. AuNPs with a protruding tip nucleic acid sequence that matches the PC-guided resonance wavelength (PCGR) allow for improvement of light harvesting throughout the surface, resulting in the enhanced collection of the incidental and scattered light. The formation of the hybrid coupling of SPR and PCGR also alters the peak reflected wavelength from the PC, which is observable for every surface-attached AuNP. This form of microscopy allows digital optical quantification and recognition of AuNP [3]. The demonstrated sandwich assay utilizes an active capture and digital counting (AC + DC) approach to measure the quantified PWV AuNP counts, which enables monitoring of circulating exosomal miRNA at a detection limit of 100 aM, and single-base mismatch selectivity in a 2 h kinetic discrimination (Figure 7) [77]. This work represents the first time that a biosensor microscopy used resonant coupling of an NP tag to a metamaterial for increased contrast to achieve a high signal-to-noise sensing system [75].

PC metamaterial surfaces combined with NP tags counted by PRAM can also be applied toward characterization of protein–protein interactions [75]. Che et al. demonstrated the AC + DC assay approach in a sandwich-based HIV capsid antigen p24 detection assay [78]. In this work, the antibody-conjugated AuNP tags are “activated” when mixed and bound to targets from the test sample. When the assay is integrated into a self-powered microfluidic chip with a PC biosensor, the “activated” AuNPs will be captured on the PC surface. The combination of the PC biosensor and the microfluidic chip bypasses the limitations of diffusion for capture of activated NP tags on the PC surface, which considerably shortens the assay time to 35 min. This technique collects the magnified change in PIV while recording the spatial distribution of the AuNP tags, which allows for single analyte tracking [66]. This approach demonstrates a 2-step PoC analysis for fast, ultrasensitive and quantification of p24 antigen detection from a single droplet of human serum with a one-million-fold dynamic range (10~10^7^ pg/mL) and a detection limit of 1 pg/mL. 

In response to the latest pandemic, PRAM-based AC + DC was adapted for COVID-19 IgG detection. Zhao et al. demonstrated a rapid, quantitative analysis of serological IgG detection against SARS-CoV-2 [79]. By simply adding the mixture of the test samples with secondary antibody (2^o^ Ab)-functionalized gold nanoparticles to the PC biosensor surface that is precoated with the COVID-19 spike protein, a sandwich immunocomplex of antigen-ab-2^o^ Ab is formed, enabling single-step, wash-free, digital resolution immunoassay with a detecting limit as low as 35 pg/mL in serum samples within 15 min. What is more, the effort of applying PRAM to PoC situations motivated the development of a portable PRAM system, utilizing a low intensity LED and a webcam-quality image sensor. The system does not require a spectrometer, which greatly suppresses the size and cost of the instrument [80].

### 3.2. Fluorescent Tags

#### 3.2.1. Photonic Crystal Metamaterials for Enhanced Fluorescence Microscopy

PC metamaterial surfaces have long been known for their capability to resonantly enhance local electromagnetic fields [23] and their ability to channel photon radiation along specific paths for enhanced emission collection efficiency. Further, photon emitters on PC surfaces can experience enhanced quantum efficiency, a shorter lifetime, and reduced blinking, as discussed in a recent review [81]. Here, we will focus on dielectric PCs for enhanced fluorescence microscopy at visible wavelengths. 

**Figure 8:**
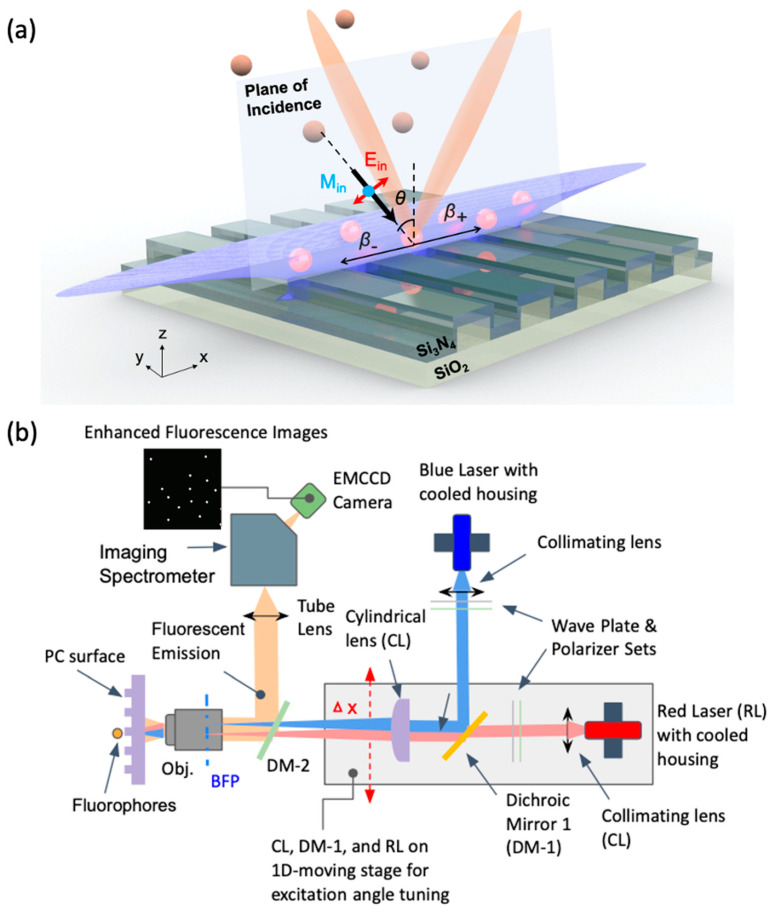
Photonic Crystal enhanced Fluorescence (PCEF). Schematic of (**a**) PC-enhanced Fluorescence platform using [82] and (**b**) objective-coupled line-scanning PCEF microscope. Two selectable incident beams are represented in blue and red illumination paths. The collection fluorescent beam paths overlap in the region between the dichroic mirror 2, PC and objective lens are represented in orange.

PC-enhanced fluorescent (PCEF) microscopy takes advantage of PC resonances associated with three phenomena (Figure 8a): (i) enhancement of the molecule excitation rate by coupling the excitation laser field into a resonance pump mode; (ii) enhancement of the extraction and collection efficiency of by redirecting the emitted light into preferred out-coupling directions. (iii) spontaneous emission engineering by modifying the photonic environment of emitters [82]. 

Enhanced excitation occurs when the laser-pump energy couples to the PC surface from specific incident angles, resulting in a strong near-field distribution across the surface that can be two orders higher than the original amplitude of incident radiation [16]. Due to an enhanced optical absorption rate, the strong optical-pump mode yields amplified output from the fluorophores. Greater Q-factor of the pump resonance leads to higher intensity near-fields; however, a moderate Q-value of 100–300 is often utilized to facilitate efficient incident light coupling.

Shown in Figure 8a, the enhanced extraction mechanism involves a spatially and spectrally biased emitted photon reradiation from near-surface fluorophores. Photons generated from fluorescent emission near the PC surface can couple to particular resonant leaky modes supported by the PC through near-field interaction. The leaky nature of the PC resonant modes results in transition of the emitted photons to propagating waves. Modulated by the PC dispersion, the emission of surface-bound fluorophores can be strongly redirected to a narrow solid angle within the numerical aperture of the imaging objective to increase the collection efficiency. 

In order to exploit multiple enhancement features simultaneously, Cunningham et al. developed the PC-enhanced fluorescent (PCEF) line-scanning microscope composed of two 70 mW solid-state lasers (Blue laser: *λ* = 450 nm and Red laser: *λ* = 637 nm) coupled to a polarizer, a half-wave plate, a movable cylindrical lens, a long pass dichroic mirror, objective lenses, filter sets, image spectrometer and EMCCD camera. Two sets of tunable lasers are switchable for exciting Quantum dots (QDs), fluorescent dye or protein at their respective absorption wavelengths. The output beam then passes through a polarizer followed by a half-wave plate, which is used to rotate the polarization to match with the PC-resonant mode to be excited. The laser beam is then focused in one axis to a line by a cylindrical lens. Incident angle θ_inc_ can be precisely tuned to excite the pump-resonate mode by shifting the focus line (Δx) on the back focal plane (BFP) of the objective. The objective lens focus laser beam on the PC surface into a line that can be translated perpendicular to the PC grating. With the PC mounted on a motorized sample stage, the system can perform line-scanning and generate enhanced fluoresce images in a large field of view (FOV). 

**Figure 9:**
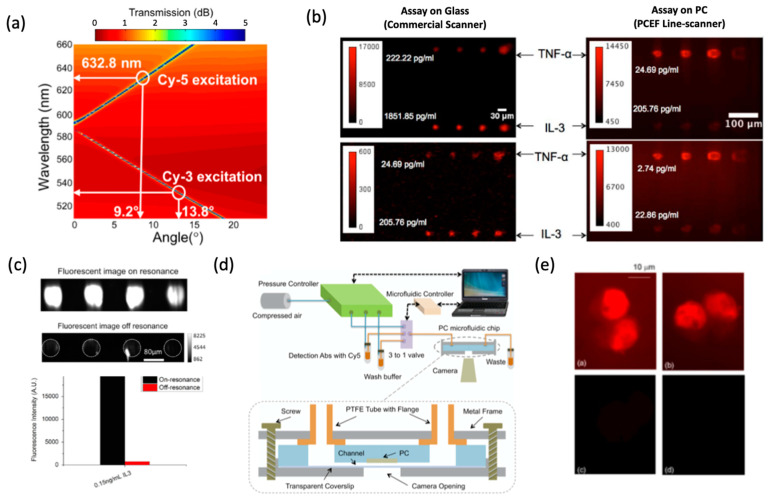
PCEF applications in bio-imaging. (**a**) PC band-diagram design for fluorescent enhancement. (**b**) Fluorescent images of micro-spots from the TNF-α and IL-3 sandwich immunoassay [83]. (**c**,**d**) Integrated microfluidic PCEF scanning system with pg/mL-level limits of detection. Reprinted with permission from Ref. [84] Copyright 2015 Elsevier B.V. (**e**) Fluorescence microscope images of labeled cells on different substrates. Top left: lateral grating plate. Top right: longitudinal grating plate. Bottom left: glass slide with coating. Bottom right: uncoated normal glass slide. Bar corresponds to 10 μm. Reprinted with permission from Ref. [85] Copyright 2015 The Optical Society.

With multiplicative enhancement effects, the PCEF system can be applied to any surface-based fluorescence assay with low detection limits, high SNR and lower-cost instrument. Being of particular interest for high-throughput, high SNR, larger FOV, lower-cost, intensity-based assays in a microarray format, PCEF was implemented for multiplexed DNA and protein detection. Mathias et al. demonstrated a 1D PC to enhance the cyanine-5 (Cy5) by a 42-fold increase in SNR when scanned on a normal glass slide with commercial microarray scanner [16]. The PC also can be engineered to generate resonances for two distinct dyes, such as Cy3 and Cy5 (Figure 9a,b) to enable differential gene expression analysis in a DNA microarray format [83]. Tan et al. adapted the PCEF detection system for multiplexed and sensitive (single digit pg/mL) detection of protein biomarkers for breast cancer [84]. The assay system consists of a plastic microfluidic cartridge with an automation system that houses the PC and offers a controllable leak-free fluid interface for point-of-care diagnostic capabilities. Tawa et al. demonstrated a fluorescent-dye-labeled enhanced cell image using subwavelength gratings covered with a thin metal layer [85]. Shown on Figure 9e, the reported fluorescence intensity was more than 20-fold higher compared to the signal from an optically flat glass substrate. 

#### 3.2.2. Metamaterial-Based Super Resolution Microscopy

**Figure 10:**
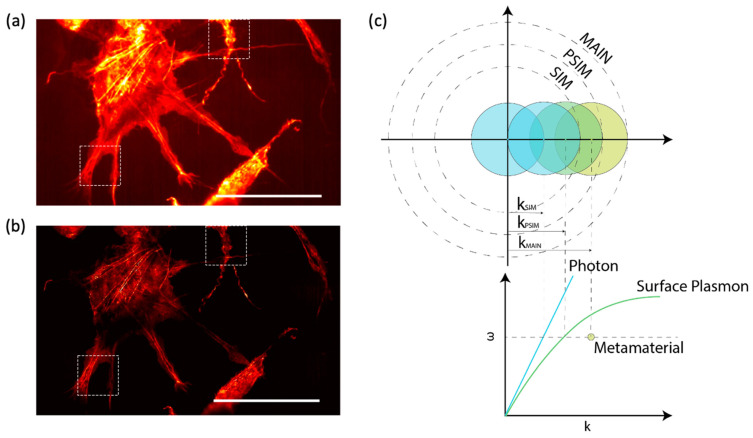
Metamaterial-based super resolution microscopy. (**a**). Diffraction limited image using conventional epifluorescence. Scale: 20 μm (**b**) speckle-MAIN image with improved spatial resolution. Reprinted from Ref. [98]. (**c**) the dispersion relation for SIM, PSIM and MAIN and their corresponding k-space shift in Fourier plane.

Fluorescence microscopy has been a key driver of numerous biological discoveries over the past few decades. However, the spatial resolution of optical microscopes was fundamentally limited by the diffraction limit of light, *λ*/(2*NA) (where NA is the Numerical Aperture of the objective lens). This implied that objects below 200 nm were not resolvable with visible light, which is critical because numerous biological processes occur below this limit. This key bottleneck was recently addressed by numerous super-resolution techniques that include stimulated emission depletion microscopy (STED) [86,87], photoactivated localization microscopy (PALM) [88,89], and structured illumination microscopy (SIM) [90,91]. Methods such as STED and PALM achieve remarkable sub-20 nm resolution but require strong light intensities that cause photodamage, while requiring lengthy acquisition times that limit observation of dynamic processes. On the other hand, SIM can presently only achieve sub-90 nm resolution, but with much faster acquisition times and minimal photodamage to the specimen. SIM achieves sub-diffraction resolution by utilizing patterned illumination, which allows imaging of higher spatial frequencies. However, since the patterned light is also diffraction-limited, the maximum possible boost in resolution can only be two times. One potential approach is to have larger spatial frequency excitation using plasmons, which have higher wave vectors when compared with photons (as shown in Figure 10c). 

Wei et al. were able to show that by using surface plasmon polaritons (SPP), they could improve the resolution by 2.7×, higher than conventional SIM methods [92]. Moreover, Ponsetto et al. showed that by using localized surface plasmon resonance they could capture even higher spatial resolution features [93]. By using an array of silver nanodiscs and tuning the angle of incidence to scan the specimen, they brought down the resolution to 75 nm. Bezryadina et al. extended this to 50 nm by using a higher NA objective and used it to produce multicolored images of the dynamics of microtubules [94]. Another promising approach is to use an imaging substrate based on hyperbolic metamaterials (HMM). These structures are made of out of a stack of thin metal and dielectrics that can improve the resolution theoretically equal to the thickness of the unit cell (~*λ*/20) [95,96]. The patterned excitation can be generated by illuminating the metamaterial from below using a patterned opaque mask. Ma et al. further described the principle of utilizing the dispersive nature of the HMM to shift the illumination pattern by using different wavelengths of excitation [97]. Moreover, Lee et al. were able to use the speckle pattern generated on the surface of the HMM to create ultra-high-resolution patterns of illumination and pushed the resolution down to 40 nm [98] (Figure 10b).

Another important aspect in super-resolution microscopy is the axial resolution for three-dimensional imaging. The conventional techniques of 4Pi-reversible fluorescent saturable optical transition (RESOLFT) [99] and interferometric PALM (iPALM) [100] have faced difficulties to achieve sub-10 nm. The recent progress in metamaterials has enabled nanometer-scale accuracy by using the distance-dependent Purcell effect. The method is based on controlling the emission rate of a fluorophore, which is directly proportional to the local density of states (LDOS) under the weak light–matter coupling condition [101,102,103]. Metamaterials allow the custom tailoring of these LDOS due to the creation of various additional photonic modes. By exploiting this effect, Lee et al. were able to achieve an axial resolution of 2.4 nm [104]. The technique worked by monitoring the rate of photobleaching, which was greatly modified by the axially varying Purcell factor. They were able to image cell membrane of HeLa cells at sub-3 nm vertical resolution by replacing the substrate with an HMM. Furthermore, they were able to extend the technique to study the changes in living HeLa cell morphology induced by epidermal growth factor [105]. 

## 4. Nanofabrication Technology for Metamaterials

**Figure 11:**
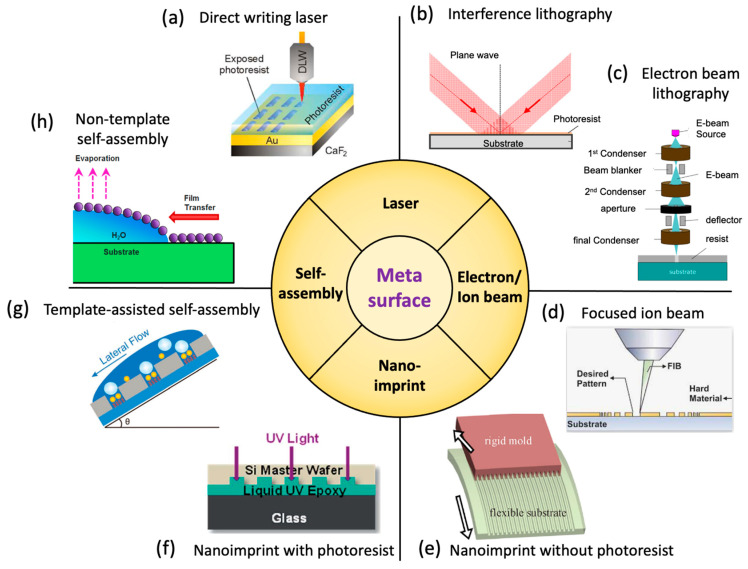
Existing methods for fabricating metamaterials. Schematic of the (**a**) Direct writing laser. Reprinted with permission from Ref. [106]. Copyright 2015 American Chemical Society. (**b**) Interference lithography. Reprinted from refs. [106,107]. (**c**) Electron beam lithography. Reprinted from Ref. [108]. (**d**) Focused ion beam. Reprinted from Ref. [109]. (**e**) Nanoimprint without photoresist. Reprinted from Ref. [110]. (**f**) Nanoimprint with photoresist. Reprinted with permission from Ref. [76]. Copyright 2014 The Royal Society of Chemistry. (**g**) Template-assisted self-assembly. Reprinted from Ref. [111]. (**h**) Non-template self-assembly. Reprinted with permission from Ref. [112]. Copyright 2013 WILEY-VCH Verlag GmbH & Co. KGaA.

The capability of producing metamaterials or metasurfaces in a low-cost and scalable fashion is essential for their practical applications. It is especially critical for their use as a substrate in microscopy, as several square millimeters of device surface area is typically required. The critical dimension of metamaterials, usually one-tenth to half of the wavelength of incident light, can range from a few nanometers to several millimeters for light spanning the ultraviolet to terahertz range. For the visible light regime, the required critical dimensions are from tens of nanometers to hundreds of nanometers, which are beyond the limit of conventional UV-lithography processes, thus requiring novel nanofabrication approaches. In this section, we summarize the existing methods into four major categories based on their mechanisms for achieving nanometer-scale features: laser-based lithography, electron/ion beam lithography, nanoimprint lithography, and self-assembly, which are listed in Figure 11. 

Microscopy applications generally require relatively large surface areas for metamaterials, while still maintaining low cost for compatibility with single-use and disposable applications that are typical for biology labware. Fortunately, metamaterials for microscopy applications do not require the sophisticated designs associated with applications such as electromagnetic cloaking. Therefore, periodic nanostructures are mostly sufficient. Since a comprehensive introduction of each technology is beyond the scope of this paper, we provide several recent comprehensive review papers on each nanofabrication technology for interested readers.

### 4.1. Laser-Based Fabrication

**Figure 12:**
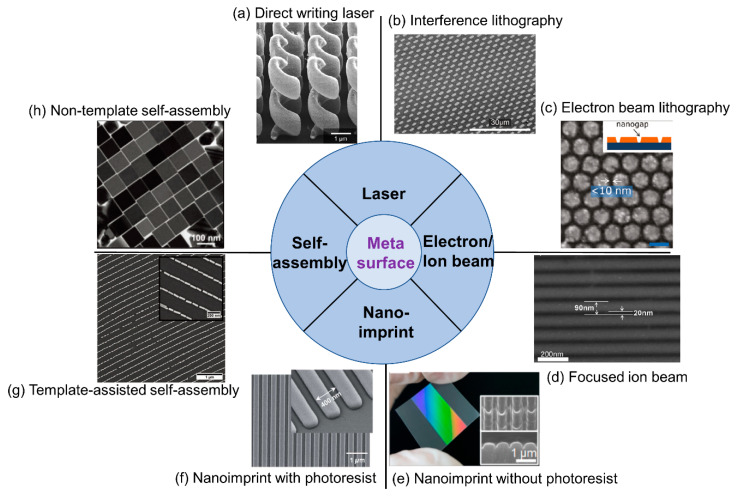
SEM images of meta-surfaces fabricated by various nanofabrication technology (**a**) Cross-sectional view of 3-D helical nanostructures fabricated by direct writing laser, Reprinted with permission from Ref. [148]. Copyright 2009 American Association for the Advancement of Science.; (**b**) A large array of rod shape by interference lithography for surface-enhanced infrared absorption. Reprinted with permission from Ref. [149]. Copyright 2014 WILEY-VCH Verlag GmbH & Co. KGaA, Weinheim. (**c**) An array of gold nanodisk array with sub-10 nm nanogaps produced by electron-beam lithography. Reprinted with permission from Ref. [116]. Copyright 2011 American Chemical Society. (**d**) An array of nanoribbons with 20 nm width produced by focused ion beam. Reprinted with permission from Ref. [126]. Copyright 2022 IOP Publishing. (**e**) A several-centimeter scale of nanopatterns of PET materials produced by nanoimprint without photoresist. Reprinted with permission from Ref. [150]. Copyright 2019 American Chemical Society. (**f**) 2-D photonic crystals fabricated by nanoimprint with photoresist. Reprinted with permission from Ref. [76]. Copyright 2014 The Royal Society of Chemistry; (**g**) an array of nanoline patterns consisting of serially connected nanorods via dip-coating using wrinkled PDMS substrates as templates. Reprinted with permission from Ref. [151]. Copyright 2014 American Chemical Society. (**h**) An array of self-assembled silver nanocubes with an average edge length of 93 ± 4 nm produced by nontemplate self-assembly. Reprinted with permission from Ref. [152]. Copyright 2016 American Chemical Society.

Laser illumination is associated with high intensity, long coherence length and a high degree of directional control, and therefore is widely used for metamaterial fabrication. Direct Writing Laser (DWL) technology integrates a laser with a pattern generator, to provide user-generated layout designs to expose a photoresist coating on a substrate with the precision comparable to the wavelength of the laser. DWL can fabricate both 2-dimensional and limited 3-dimensional metamaterials with pattern features extending from visible wavelengths to terahertz for multiple applications, such as substrates for surface-enhanced infrared absorption [106] and invisibility cloaks [113]. As shown in Figure 12a, the 3-dimensional helical photonic metamaterial is fabricated by DWL to control the circular polarization of light. However, the precision of DWL is limited to λ/NA, where *λ* is the wavelength of the laser, and NA is the numerical aperture of the lens used to focus the beam. Due to lengthy writing times, DWL provides only low throughput, and thus wafer-scale metamaterials may be prohibitively expensive. 

Another fabrication technology based on laser exposure is interference lithography, which is often also called holographic lithography. Interference lithography uses interference patterns between two coherent lasers to expose photoresists to generate periodic features. Compared with DWL, the precision of interference lithography is improved to $\frac{\lambda /2}{\sin\left(\frac{\theta }{2}\right)}$, where *λ* is the wavelength and *θ* is the angle between the two interfering waves. This method includes some important benefits of DWL, such as the ability to choose among many periodic lattice configurations (i.e., linear, square, hexagonal) and the ability to pattern three-dimensional structures [114,115,116]. Interference lithography can be performed on large-area glass or silicon substrates, in either single-shot exposure of the entire wafer or sequential exposure of an array of tiles [117]. As shown in Figure 12b, a centimeter-scale, surface-enhanced infrared absorption substrate is fabricated through interference lithography, which demonstates preparation of a metasurface with large surface area. Interference lithography can be achieved with either wavefront-divided Lloyd’s mirror illumination [118] or amplitude-divided multibeam illumination [114]. The amplitude-divided multibeam approach is preferred for producing large-area and high-quality nanopatterns. One of the challenging issues for laser interference lithography is that nanometer-scale mechanical vibration can induce displacement errors that can cause the interference condition to change, resulting in pattern degradation. An actively stabilized, phase-locking mechanism was added to render a so-called fiberoptic interference lithography (FOIL), which exhibits improved reliability and uniformity over a long laser exposure duration [119] capable of producing periodic nanopatterns over a 3-inch wafer scale [120]. Two recent review papers dedicated to interference lithography are listed here [121,122].

### 4.2. Electron/Ion Beams

Electron and ion beam patterning technology has very high precision, and the ability to generate features as small as several nanometers because the diffraction limit for charged particles is much smaller than ultraviolet wavelengths. However, the scanning speeds for electron/ion beam exposure are also strongly limited by the low dose rate compared to the laser, reducing their suitability in scalable nanomanufacturing. However, these forms of lithography are excellent for producing small-area proof-of-concept designs [115,123,124] or for producing “master” structures that can be used for various forms of replica molding such as nanoimprint, to be introduced in Section 4.3. 

Based on the specific mechanism, there are three types of lithography in this category. The first type is to use the electron beam or ion beam to expose specially formulated resists such as electron beam lithography (EBL) and ion beam lithography (IBL). A second approach is focused ion-beam milling (FIBM) wherein ions are accelerated to bombard on the material to physically etch nanostructures without the use of resist. The third approach is beam-induced deposition, including focused electron-beam-induced deposition (FEBID) and focused ion-beam-induced deposition (FIBID). In these approaches, reactive ion gases are provided while beams locally induce the reactive gases to form solid nanostructures from materials including carbon and platinum. 

Using these methods, ultrasmall nanogaps between metallic patterns can be produced that can generate enhanced electromagnetic fields [116]. As shown in Figure 12c, direct and reliable patterning of plasmonic nanostructures with sub-10-nm gaps EBL has been demonstrated. In addition, graphene plasmons extensively utilize electron or ion-beam lithography [125,126] for generating localized graphene plasmons with ultrasmall structures. Direct patterning of graphene nanoribbons with 20 nm width can be achieved by FIB patterning. The advantages of electron/ion beam methods are the small resolution ~10 nm and capability for producing arbitary shapes of nanopatterns. 

Review papers about electron-beam lithography [127,128] and ion-beam lithography [129] are listed here. Figure 12c shows an array of gold nanodisks with sub-10 nm nanogaps produced by EBL, and Figure 12d is a SEM image of an array of nanoribbons with 20 nm width produced by FIB patterning.

### 4.3. Nanoimprint

Nanoimprint lithography replicates metamaterial nanostructures from a master mold template onto a target substrate through processes that include thermally assisted mechanical deformation, UV-cured polymers or chemical transfer. Since its invention in 1990 for the thermal printing modality, it has developed into various types such as thermal nanoimprinting [130], ultraviolet nanoimprinting [131], hard imprinting [132], and roll-to-roll nanoimprinting [133]. Nanoimprinting can produce large area samples at low cost, and has the combined advantages of high throughput and high precision. However, nanoimprint requires a custom nanopattern template, which is usually expensive. On the other hand, since the master mold can be utilized many times, the cost for each part is significantly reduced. 

Nanoimprint can utilize photoresist as a template or alternatively can be performed directly without photoresist. Nanoimprinting with photoresist has the advantages of high resolution and low deformation of the structure after sufficient hardening by curing. For example, duplication in production of photonic crystal with high precision is demonstrated using ultraviolet nanoimprint, as shown in Figure 12f. However, polymer residue can be transferred to the device, requiring additional processes to remove contamination. Non-photoresist nanoimprinting utilizes “hard” materials such as metal, silicon or glass to transfer patterns to the workpiece, generally “soft” materials such as plastic substrate, which is shown in Figure 12e. In this process, high temperature and/or high pressure is usually needed. This method can produce the desired pattern directly on the workpiece, albeit with limitations on the material that the final structure is composed of. Based upon these approaches, photonic crystal biosensors [76], negative refractive index materials, [134] and meta-absorbers [135] have been demonstrated. Two comprehensive review papers on nanoimprint lithograhy are listed below for readers [136,137].

### 4.4. Self-Assembly

Self-assembly is a bottom-up nanofabrication method that uses basic building blocks such as nanoparticles, nanocrystals, block-coplymers, and nucleic acid molecules to spontaneously form nanostructures based on the interaction between those building blocks. The interaction between these building blocks can be controlled through surface charges, hydrophobicity, hydrophilicity, and chemical functionality [138,139]. The implementation of self-assembly includes drop-casting [140], spin-coating [141], and capillary force-assisted particle assembly [142]. Self-assembly of metamaterials can be adapted toward large surface areas suitable for microscopy. The disadvantage of self-assembly methods is that the nanostructure shapes that can be produced are limited to simple and periodic geometries.

Self-assembly is capable of producing large-area nanopatterns and is capable of extenting toward 3-D nanostructures. Self-assembled metamaterials have been demonstrated that comprise gold nanocubes [143], silver nanorods [144], and polystyrene nanospheres [145]. Figure 12e is a typical example for non-template self-assembly of silver nanocubes. Moreover, self-assembly can be combined with other fabrication methods such as etching and evaporation to produce nanostructures with inverse opal structures that enhance Raman scattering signals [146]. Template-assisted self-assembly can partly overcome the drawback of periodic geometries. By preparing the designed structure on the substrate, nanomaterials tend to aggregate in the cupped patterns during the process of self-assembly, as shown in Figure 12g.

Figure 12e shows several centimeter-scale nanopatterns of PET materials produced by nanoimprint without photoresist, and Figure 12f shows 2-D photonic crystals fabricated by nanoimprint with photoresist. Existing literature summarizing self-assembly methods is listed here [138,147].

Generally speaking, 2-D structured metamaterials such as nanohole and nanodisks require more stringent control of positioning than 1-D stuctured metamaterials. For example, self-assembly could produce 1-D nanostructures but it is very challenging to produce 2-D nanostructures with uniform gaps. Therefore, most 2-D structured metasurfaces are either produced by EBL or by nanoimprint with EBL-produced master molds. Moreover, for applications requiring high Q-factor metamaterials, EBL is preferred for its precision in both nanostructure definition and precise positioning. Laser interference lithography can produce metasurfaces with sufficient uniformity for applications demanding large areas.

## 5. Discussion and Outlook

The preceding discussion shows that, by utilizing their capacity to capture and concentrate the electromagnetic energy associated with light into small volumes while also interacting with broad classes of photon emitters to modulate their properties and to enhance collection efficiency, photonic metamaterials are demonstrating great promise as a substrate for several forms of microscopy. Metamaterials are capable of increasing the absorption cross-section and scattering cross-section for molecules, viruses, and nanoparticle tags, enabling each to be observed digitally as individual objects with high signal-to-noise ratio. Meanwhile, photon emitters engage with hybrid interactions with the metamaterial when in proximity to alter the fundamental properties of lifetime and blinking on/off time ratio for more favorable detection. Importantly, metamaterial resonances must be designed to coincide with the absorption wavelengths and emission wavelengths of plasmonic nanoparticles, semiconductor quantum dots, and molecules. Through design of metamaterials that simultaneously provide multiple resonances, adjustment of incident angle in an appropriately designed instrument and through the utilization of tunable-wavelength illumination sources, the enhancements can be easily applied to broad classes of molecules and nanoparticles. Because the phenomena of enhanced excitation (or absorption) and enhanced extraction operate by independent mechanisms, their effects are shown to multiply when used together, resulting in thousands-fold enhancements of signal-to-background, particularly for observation of individual objects. In the context of super-resolution fluorescence microscopy, photonic metamaterials are utilized in a different way, where their periodic modulation naturally generates spatially periodic and surface-confined excitation.

The capability for imaging-based detection of enhanced effects on metamaterial surfaces is driving several applications in biomolecular diagnostics, viral-load monitoring, and cell imaging. While PRISM-based imaging utilizes label-free detection and counting of individual proteins and viruses (either surface-captured or freely diffusing in solution) and the ability to estimate their size through their image contrast, functionalized nanoparticle tags (such as AuNPs, AuNRs, and quantum dots) used to label biomarker molecules are enabling ultrasensitive and ultraselective detection of nucleic acids and proteins with quantitative accuracy over a broad dynamic range, while using simple inexpensive instruments. Likewise, metamaterials utilized for super-resolution imaging, darkfield imaging, and phase-contrast imaging are providing greater contrast while simplifying the detection instrumentation.

Future developments in this area are certain to incorporate more advanced metamaterial designs that will include hyperbolic metamaterials, 2-dimenstional structures, and quasi-2-dimensional structures that will incorporate features beneath the image plane. Novel materials will likely play exciting roles by incorporating porosity, tunability, and transparent electrodes. We anticipate that photonic metamaterials will become compatible for use within existing microscope instruments, while others designed for optimal performance will require specially configured systems that feature tunable wavelength illumination sources and/or the ability to modulate the incident angle. While many of the advances reviewed here are in the realm of academic research, it is likely that commercially available products will become more broadly available in the next 5 years. The authors of this manuscript have pursued metamaterial-enhanced microscopy using photonic-crystal surfaces, which offer combined advantages of large-scale fabrication, moderately high quality factor, simple mode structure, and high reflection efficiency at the resonant wavelength. As this review shows, a broad set of surface structures, materials, and phenomena are contributing to a rich field of research around which a growing community is participating. The community is motivated not only by novel and interesting optical phenomena, but also by the impactful applications that are enabled by enhancing the interactions between light and nanomaterials. Our review highlighted commercially important applications in molecular diagnostics, pathogen detection, and life science research, which represent only a small fraction of the potential opportunities in food safety, environmental monitoring, pharmaceutical development, point-of-care health diagnostics, biodefense, and agriculture.
